# Spatial and Temporal Trends of SARS-CoV-2 RNA from Wastewater Treatment Plants over 6 Weeks in Cape Town, South Africa

**DOI:** 10.3390/ijerph182212085

**Published:** 2021-11-17

**Authors:** Renée Street, Angela Mathee, Noluxabiso Mangwana, Stephanie Dias, Jyoti Rajan Sharma, Pritika Ramharack, Johan Louw, Tarylee Reddy, Ludwig Brocker, Swastika Surujlal-Naicker, Natacha Berkowitz, Mokaba Shirley Malema, Sizwe Nkambule, Candice Webster, Nomfundo Mahlangeni, Huub Gelderblom, Mongezi Mdhluli, Glenda Gray, Christo Muller, Rabia Johnson

**Affiliations:** 1 Environment & Health Research Unit, South African Medical Research Council (SAMRC), Tygerberg 7505, South Africa; angie.mathee@mrc.ac.za (A.M.); shirley.malema@gmail.com (M.S.M.); sizwe.nkambule@mrc.ac.za (S.N.); candice.webster@mrc.ac.za (C.W.); nomfundo.mahlangeni@mrc.ac.za (N.M.); 2 Environmental Health Department, Faculty of Health Sciences, University of Johannesburg, Johannesburg 2092, South Africa; 3 Biomedical Research and Innovation Platform (BRIP), South African Medical Research Council (SAMRC), Tygerberg 7505, South Africa; noluxabiso.mangwana@mrc.ac.za (N.M.); stephanie.dias@mrc.ac.za (S.D.); jyoti.sharma@mrc.ac.za (J.R.S.); pritika.ramharack@mrc.ac.za (P.R.); Johan.Louw@mrc.ac.za (J.L.); christo.muller@mrc.ac.za (C.M.); rabia.johnson@mrc.ac.za (R.J.); 4 Department of Microbiology, Stellenbosch University, Stellenbosch 7600, South Africa; jhlbrocker@gmail.com; 5 Department of Biochemistry and Microbiology, University of Zululand, KwaDlangezwa 3886, South Africa; 6 Biostatistics Unit, South African Medical Research Council (SAMRC), Tygerberg 7505, South Africa; tarylee.reddy@mrc.ac.za; 7 Scientific Services, Water and Sanitation Department, City of Cape Town Metropolitan Municipality, Cape Town 8000, South Africa; swastika.surujlalnaicker@capetown.gov.za; 8 Community Services and Health, City Health, City of Cape Town, Hertzog Boulevard, Cape Town 8001, South Africa; natacha.berkowitz@capetown.gov.za; 9 COVID-19 Prevention Network (COVPN), Fred Hutchinson Cancer Research Center, Seattle, WA 98109, USA; hgelderb@fredhutch.org; 10 Office of the President, South African Medical Research Council, Tygerberg 7050, South Africa; mongezi.mdhluli@mrc.ac.za; 11 Chief Research Operations Office, South African Medical Research Council, Tygerberg 7050, South Africa; glenda.gray@mrc.ac.za; 12 Centre for Cardio-Metabolic Research in Africa, Division of Medical Physiology, Faculty of Medicine and Health Sciences, Stellenbosch University, Stellenbosch 7600, South Africa; 13 Discipline of Pharmaceutical Sciences, School of Health Sciences, University of KwaZulu-Natal, Westville Campus, Durban 4001, South Africa

**Keywords:** SARS-CoV-2, COVID-19, wastewater, environmental epidemiology, South Africa

## Abstract

Recent scientific trends have revealed that the collection and analysis of data on the occurrence and fate of SARS-CoV-2 in wastewater may serve as an early warning system for COVID-19. In South Africa, the first COVID-19 epicenter emerged in the Western Cape Province. The City of Cape Town, located in the Western Cape Province, has approximately 4 million inhabitants. This study reports on the monitoring of SARS-CoV-2 RNA in the wastewater of the City of Cape Town’s wastewater treatment plants (WWTPs) during the peak of the epidemic. During this period, the highest overall median viral RNA signal was observed in week 1 (9200 RNA copies/mL) and declined to 127 copies/mL in week 6. The overall decrease in the amount of detected viral SARS-CoV-2 RNA over the 6-week study period was associated with a declining number of newly identified COVID-19 cases in the city. The SARS-CoV-2 early warning system has now been established to detect future waves of COVID-19.

## 1. Introduction

Wastewater-based epidemiology has played an important role in the development of early warning systems (EWS) for various enteric viruses, including poliovirus, norovirus and hepatitis [1]. In the current COVID-19 pandemic, wastewater-based epidemiology has shown the potential to provide a platform for SARS-CoV-2 surveillance. SARS-CoV-2 RNA has been detected in the feces of both symptomatic and asymptomatic cases [2] and research in many countries, including France, the Netherlands and the United States of America (USA), has shown a correlation between SARS-CoV-2 RNA viral loads in wastewater and COVID-19 clinical case data [3,4]. For example, a study in the Netherlands detected SARS-CoV-2 RNA in wastewater even when the COVID-19 prevalence was low [4]. Such findings have provided the evidence-base for the Netherlands to incorporate sewage surveillance as an EWS within its national response to COVID-19 monitoring and other countries, including Australia, Germany, New Zealand and the USA, are taking similar steps. According to the World Health Organization (WHO) [5], it has been demonstrated that wastewater-based epidemiology has the potential to be used for monitoring COVID-19 prevalence and temporal trends, however it is necessary to pilot this approach in low- and middle-income (LMIC) settings to demonstrate its added value to support clinical surveillance. An effective wastewater-based epidemiology system may prove critical where health systems infrastructures, testing systems, personal protective equipment (PPE) and human resource capacities are constrained.

The City of Cape Town, in the Western Cape Province, is one of eight metropolitan municipalities in South Africa, with approximately 4 million inhabitants. It is one of the wealthiest cities in Africa but is also recognized as having one of the highest income inequalities globally. This inequality extends to health care access and living conditions, leading to variations in the transmission and spread of COVID-19 within the metropolis. As such, wastewater surveillance within the City of Cape Town can assist in unbiased tracking of COVID-19 at the sub-city scale to determine potential localized outbreaks. The first COVID-19 case in the Western Cape was reported on 11 March 2020 and the confirmed cases gradually increased after that point; from 1 May to 1 June 2020, the number increased from 226 cases to 1668 cases, respectively [6]. In early July 2020, the Western Cape emerged as the epicenter of COVID-19 in South Africa and at the time carried the highest proportion of COVID-19 cases (35%) as well as related deaths (64%) in the country [7]. To coincide with the COVID-19 peak in the Western Cape, this study undertook wastewater sampling in 23 WWTPs in the City of Cape Town and determined the spatial and temporal SARS-CoV-2 trends over a six-week period.

## 2. Materials and Methods

### 2.1. Wastewater Sampling

From 6 July 2020, one 500 mL untreated influent wastewater grab sample was collected at the raw inlet after coarse screening from each of the 23 wastewater treatment plants (WWTPs) in the City of Cape Town. Samples were collected once a week on a Monday with sample collection occurring at a similar time each week over a 6-week period. Two of the WWTPs (namely, Millers Point and Oudekraal) served as controls as they are situated on nature reserves and do not serve residential populations. A random computer-generated number was assigned to each WWTP and the laboratory staff were blinded to the incoming samples. All samples were transported to the laboratory on ice and processed immediately for the extraction of RNA, after which it was stored at −80 °C for subsequent quantitative real-time polymerase chain reaction (qRT-PCR).

### 2.2. Sample Concentration and RNA Extraction

A modified method described by Peccia et al. [8] and optimized by Johnson et al. [9] was used in order to extract the total RNA using the Qiagen RNeasy^®^ PowerSoil^®^ Kit, as per the manufacturer’s instructions (Qiagen, Hilden, Germany). Briefly, 100 mL of influent wastewater was spun down at 2500× *g* for 20 min, whereafter 2–5 mL of the resultant supernatant pellet was added to a 15 mL PowerBead^®^ Tube containing a lysis buffer to inactivate the virus and stabilize the viral RNA. Thereafter, the sample was homogenized and phase-separated using an equal volume of phenol/chloroform and the upper aqueous phase was transferred to a new 15 mL tube and mixed with the required buffers as supplied within the RNeasy PowerSoil kit. The RNA isolation sample was then transferred to the RNeasy JetStar Mini Column to elute out the bound RNA before centrifugation at 13,000× *g* for 15 min. The resultant pellet was dried and dissolved in a final volume of 50 µL of ribonuclease-free water. The quantity and quality of the total RNA was measured by spectrophotometry using the NanoDrop^®^ ND-1000 instrument (Nanodrop Technologies, Wilmington, NC, USA). In the absence of a surrogate, a clinical SARS-CoV-2 positive nasal swab sample with known viral copies was used to spike a wastewater sample in order to investigate the efficiency of the extraction method.

### 2.3. Quantitative Real-Time Polymerase Chain Reaction Analysis

The Centers for Disease Control and Prevention (CDC) approved quantitative real-time polymerase chain reaction (qRT-PCR) N1 and N2 primer/probe assays (Table 1) [10], which were purchased from Whitehead Scientific (Integrated DNA Technologies, Coralville, USA) and used for the detection of SARS-CoV-2 viral RNA in wastewater samples. Both N1 and N2 primer/probe sets aligned 100% with the N-protein of the SARS-CoV-2 strain. For qRT-PCR positive control and viral RNA copy number quantification, a 1:10 fold serial dilution was made with assays 2019-nCoV-N-Positive plasmid control, which was supplied at 200,000 copies/µL (Qauntabio, Beverley, MA, USA). For qualitative and quantitative analysis detection of SARS-CoV-2 viral RNA, a one-step qRT-PCR reaction was performed using the iTaq^TM^ Universal Probes One-Step Reaction kit, according to the manufacturer’s instructions (Bio-Rad Laboratories, Richmond, CA, USA). Briefly, 1 µL of 0.2 µg/µL of total RNA was used for the qRT-PCR reaction in a final volume of 10 µL, using the QuantStudio™ 7 Flex Real-Time PCR System (ABI instrument, Life Technologies, Carlsbad, USA). All reactions were performed in technical duplicate and a template control was included for each experimental run. To minimize potential contamination, RNA extraction and qRT-PCR were performed in separate laboratories.

### 2.4. Statistical Analysis

The SARS-CoV-2 copies per milliliter of wastewater were described using summary statistics. The Shapiro–Wilk test was used to test the normality of SARS-CoV-2 copies per milliliter. Spearman’s rank correlation was used to determine the correlation between N1 and N2 primers. The signed rank test was used to test whether there was a significant change in SARS-Cov-2 RNA signals between weeks 1, 3 and 6. At week 1, quartiles estimated from the average of N1 and N2 were used to form the four RNA signal categories (category 1 with the lowest and category 4 with the highest RNA signal) which formed the basis for the maps presented. Additionally, a Spearman’s rho correlation analysis was performed on the wastewater and clinical cases.

### 2.5. Spatial Data

Suburb shapefiles were obtained from the City of Cape Town’s open data portal. Coordinates for each WWTP were collected using a handheld GPS and verified using Google Earth. All maps were produced using ArcGIS 10.6.1. (ESRI, Durban, South Africa). The WWTP catchment areas (comprising of 753 suburbs) were joined to the corresponding WWTPs.

## 3. Results

WWTP capacity ranged from 0.03 Ml/day to 200 Ml/day (Oudekraal and Cape Flats, respectively). The WWTP site characteristics are presented in Supplementary Table S1. A total of 138 grab samples, one per WWTP, were collected weekly over 6 weeks, of which 12 samples were collected from the two control sites. A 15% recovery efficiency of the extraction procedure was in line with previously published studies [11,12,13]. There was a strong correlation between viral RNA copies/mL for N1 and N2 primers (r = 0.897, *p*-value < 0.001). Samples from both control sites (Millers Point and Oudekraal WWTP) contained undetectable viral RNA loads over the 6-week period. Week 1 had the highest overall (median) viral RNA signal (9200 RNA copies/mL). The extremely high SARS-CoV-2 RNA signal recorded in week 1 at the Mitchells Plain WWTP (37,486,080 copies/mL) was flagged and re-analysis in the laboratory confirmed this result. There were a median decrease of 4779.5 RNA copies/mL between weeks 1 and 3 (*p*-value 0.001) and a median decrease of 3592 copies/mL between weeks 3 and 6 (*p*-value 0.0001). The median SARS-CoV-2 RNA for weeks 2, 4 and 5 were 3448, 4032 and 583 copies/mL, respectively.

**Figure 1:**
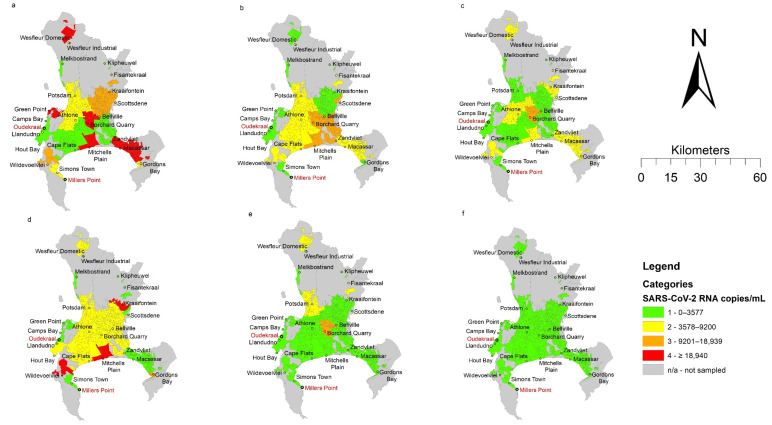
Spatial representation of SARS-CoV-2 RNA/mL in wastewater from 23 wastewater treatments plants and related catchment areas (suburbs) in the City of Cape Town at 6 sampling timepoints: (**a**): 6 July 2020 (week 1); (**b**): 13 July 2020 (week 2); (**c**): 20 July 2020 (week 3); (**d**): 27 July 2020 (week 4); (**e**): 3 August 2020 (week 5); (**f**): 10 August 2020 (week 6).

**Figure 2:**
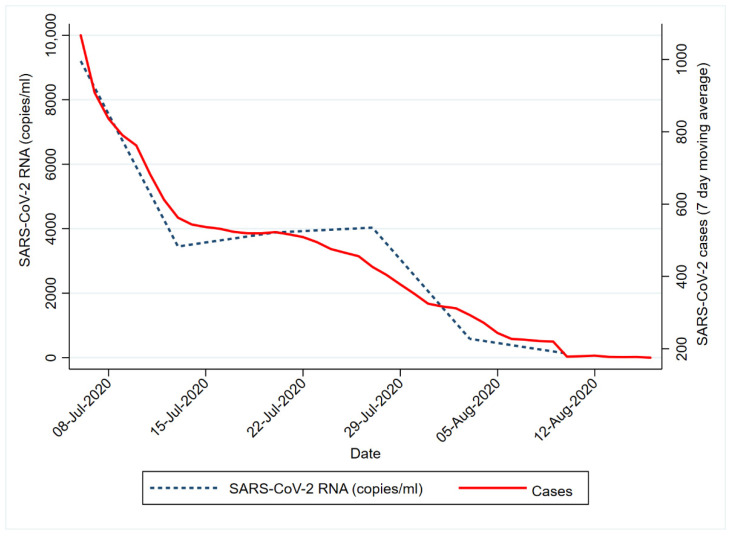
Number of confirmed new COVID-19 cases in the City of Cape Town from the period of 6 July 2020 to 10 August 2020. Data retrieved from the COVID-19 Dashboard from the Western Cape Provincial Government [6].

The spatial representation (Figure 1) shows the changing trend in SARS-CoV-2 RNA signals over time in relation to the starting time point (week 1). Based on the Inter Quartile Ranges (IQR), the RNA signals observed from the wastewater samples progressively decreased from week 1 to week 6. It must be noted that despite the decreased SARS-CoV-2 RNA load detected in most of the WWTPs, areas serviced by the Mitchells Plain and Borcherds Quarry WWTP had higher viral loads. Notably, both Mitchell’s Plain and Borcherds Quarry WWTPs were ‘hotspots’ (i.e., higher signal areas) in week 1. Additionally, the reduction in SARS-CoV-2 RNA corresponded with the decline in COVID-19 clinical cases over the same period (Figure 2). This was further confirmed by the Spearman’s rank test, which showed a significant positive correlation (r = 0.83; *p* = 0.0416) between the reported clinical cases and the SARS-CoV-2 viral RNA in the wastewater.

## 4. Discussion

The COVID-19 pandemic has had devastating societal and economic impacts on South Africa [14]. In this study, we determined spatial and temporal trends of SARS-CoV-2 in wastewater as the basis for a COVID-19 early warning system. The overall trends show the declining SARS-CoV-2 RNA signal that corresponds with the declining COVID-19 case numbers in Cape Town as reported by the Western Cape Provincial Government. Arora et al. [15] reported the presence of SARS-CoV-2 RNA in the wastewater over a period of three weeks in Jaipur City, India. Low SARS-CoV-2 RNA signal in wastewater corresponded to the low number of positive COVID-19 cases in the city. In Brazil, researchers monitored the SARS-CoV-2 RNA signal in wastewater for 41 weeks in the city of São Paulo and observed a positive correlation between the RNA signal in the wastewater and clinical cases [16]. Our results are consistent with these studies. In our study, untreated, influent wastewater samples were collected from the inlet of WWTPs, which other studies have demonstrated is the preferred sample type for investigating changes in SARS-CoV-2 RNA signals and correlations between clinical cases and hotspot identification [17,18].

The heterogeneous RNA signals within the City of Cape Town highlight the importance of temporal trends at a sub-city scale, especially for densely populated cities. A study in south-eastern Virginia (USA) suggested that as the COVID-19 pandemic wanes, it is likely that communities will see an increased incidence of small, localized outbreaks which can be detected by water-based epidemiology [19]. In South Africa, increased local transmission of the virus has been noted in the second and third waves, as well as following local super-spreader events [20,21]. The surveillance of suburbs allows for authorities to pin-point small outbreaks which for South Africa and other resource-constrained countries can guide targeted interventions and timeous public health responses [22].

In March of 2021, The European Commission issued a recommendation for nationwide wastewater monitoring system for SARS-CoV-2 surveillance as a complementary approach to monitor the spread of COVID-19 in all its member states [23]. Owing to reports of the detection and tracking of the virus in wastewater by several European countries in 2020, all EU member states were called to establish a wastewater surveillance system by October 2021. High-income countries with well-equipped sanitation systems, such as Finland [24], the Netherlands [25] and the USA [26], have established nationwide COVID-19 wastewater monitoring programs. Wastewater surveillance has shown to be a useful alternative and low-cost tool for population-level screening in these countries.

In LMICs, several studies have detected the presence of SARS-CoV-2 in wastewater including Argentina [27], Brazil [28] and India [29]. To our knowledge, this is the first study in an African setting that reports the spatial and temporal trends of the SARS-CoV-2 RNA signal in wastewater covering an entire Metro. This study demonstrates how wastewater monitoring of SARS-CoV-2 can be of benefit for and can support clinical testing, identifying hotspots, informing preparedness and monitoring the effectiveness of response measures brought about by public health officials.

The SARS-CoV-2 RNA signals in the study are reported as the virus concentration without normalization due to uncertainties in readily available information about flow rate and population size estimates. As highlighted by Hou et al. [30], census data is often outdated and does not accommodate for population mobility in cities, while the design capacity of a WWTW is not reflective of the real time load in a sewerage system. Moreover, Feng et al. [31] found that normalization to WWTP characteristics had a minimal impact when correlating SARS-CoV-2 RNA in wastewater to clinical cases. Given these uncertainties, the limited availability of such information in the study and the South African context, future research can explore normalization using a human fecal marker, such as pepper mild mottle virus.

At the time of sampling, South Africa was in lockdown level three, which indicated the most moderate of the five severity levels. Although the first wave of COVID-19 cases has since passed, there has been a resurgence of COVID-19 clusters and outbreaks, with the country currently experiencing a third wave [32]. A rapid public health response is therefore critical to prevent further morbidity and mortality, as health care systems in South Africa have come under strain. Moreover, limited and biased testing data may mask the estimates of COVID-19 infections, which are critical to informing public health responses [33,34]. The SARS-CoV-2 viral load in wastewater represents both asymptomatic and symptomatic shedding in a specific geographic location, thus providing a less biased dataset which can be used to assess infection prevalence [19]. Results from this study further support the need for prolonged wastewater surveillance as an additional strategy to current tracing methods employed to mitigate COVID-19 spread. Nonetheless, this study has a few key limitations. The wastewaters samples were collected using the grab sampling technique. Therefore, the results presented for each week are reflective of one time point. However, the routine sampling strategy adopted in this study allowed for weekly comparisons of concentrations to be made at each wastewater treatment plant. Furthermore, the SARS-CoV-2 variants of concern (VOC) have played an integral part in driving the pandemic. SARS-CoV-2 VOC testing was not undertaken in this study; however, our current and future wastewater surveillance studies now include VOC testing.

## 5. Conclusions

In conclusion, we report the detection of SARS-CoV-2 RNA in the wastewater of the City of Town’s WWTPs. We identified an overall decrease in the amount of detected viral RNA over the 6-week study period, associated with a declining number of newly identified COVID-19 cases. This is the first study on the African continent to determine spatial and temporal trends of SARS-CoV-2 spanning an entire metropolitan city. As South Africa has declining COVID-19 case numbers, ongoing wastewater surveillance is an economical early warning system that can guide public health responses. On the basis of these study results and the feasibility of trend detection, a pilot early warning system is underway in various cities across South Africa to identify potential COVID-19 resurgences at the sub-city scale.

## Figures and Tables

**Table 1:** Thermal cycling protocol and primers and probes used in the study.

Organism	Target	Assay Name	Target	Part Number	Product Number	Cycling Parameters	References
SARS-CoV-2	N protein	2019-nCoV CDC	N1 primer/probe	RV202001 RV202015	10006606	50 °C–10 min 95 °C–3 min PCR: 40 Cycles 95 °C–15 s 60 °C–60 s	[8,10]
			N2 primer/probe	RV202002 RV202016	10006606		

## Data Availability

Data available from author upon reasonable request.

## Supplementary Materials

The following are available online at https://www.mdpi.com/article/10.3390/ijerph182212085/s1, S1: Wastewater treatment plant characteristics.
