# Supplementary material for: Spatial and Temporal Trends of SARS-CoV-2 RNA from Wastewater Treatment Plants over 6 Weeks in Cape Town, South Africa
Source: Int J Environ Res Public Health. 2021 Nov 17;18(22):12085. doi: 10.3390/ijerph182212085 (PMC8618134; doi:10.3390/ijerph182212085)
Supplement: Supplementary file 1 [file ijerph-18-12085-s001.zip › ijerph-1350742-supplementary.pdf]

International Journal of
Environmental Research and Public Health

Table S1. Wastewater treatment plant characteristics (1).

Wastewater Treatment Plant	Capacity (Ml/day)	Plant type
Athlone	105	Activated sludge
Bellville	54.6	Activated sludge
Borcherds Quarry	35	Activated sludge
Camps Bay	5.5	Sea outfall
Cape Flats	200	Activated sludge
Fisantekraal	24	Activated sludge
Gordons Bay	3.1	Activated sludge
Green Point	40	Sea outfall
Hout Bay	9.8	Sea outfall
Klipheuwel	0.07	Rotating bio disc
Kraaifontein	17.5	Activated sludge
Llandudno	0.28	Rotating bio disc
Macassar	38	Activated sludge
Melkbosstrand	5.4	Activated sludge
Millers Point	0.06	Rotating bio disc
Mitchells Plain	45	Activated sludge
Oudekraal	0.03	Rotating bio disc
Potsdam	47	Activated sludge
Scottsdene	12.5	Activated sludge
Simons Town	2.5	Bio filters
Wesfleur Domestic	8	Activated sludge
Wesfleur Industrial	6	Activated sludge
Wildevoelvlei	14	Activated sludge
Zandvliet	72	Activated sludge

1. Water and Sanitation department of the City of Cape Town. Water Services and the Cape Town Urban Water cycle. 2018.
